# Application of the Lower Extremity Functional Scale and Its Correlation with Lymphedema Health-Related Quality of Life on Lower Limb Filarial Lymphedema Patients

**DOI:** 10.1089/lrb.2019.0045

**Published:** 2020-06-16

**Authors:** De Britto Lourduraj, Satish Prasad Barnawal, Kamaraj Pattabi, Vijayalakshmi Gnanasekaran, Anbusivam Sadhasivam, K. Supriya Vinod, Deep Sharma, Yuvaraj Jayaram

**Affiliations:** ^1^ Unit of Clinical Epidemiology and Chemotherapy, Vector Control Research Centre, Indian Council of Medical Research (ICMR), Pondicherry, India.; ^2^ Department of Orthopaedics, JIPMER, Pondicherry, India.; ^3^ Division of Epidemiology & Biostatistics, National Institute of Epidemiology-ICMR, Chennai, India.; ^4^ College of Physiotherapy, Mother Theresa Post Graduate and Research Institute of Health Sciences, Pondicherry, India.

**Keywords:** filariasis, elephantiasis, lymphedema, microfilaria, LEFS, quality of life

## Abstract

***Introduction:*** This study carried out as a part of the lymphedema (LE)—osteoarthritis project to know the feasibility and applicability of lower extremity functional scale (LEFS) and LE health-related quality of life (LEHRQoL) among filarial LE patients of the lower extremity.

***Materials and Methods:*** Following inclusion and exclusion criteria 30 LE patients and 30 controls were recruited in the study. After obtaining informed written consent, Tamil version of the two “self-reporting assessment tools” LEFS and LEHRQoL were applied to all the participants by two examiners independently. Feasibility was assessed by the time schedule. Internal consistency and the correlation between two examiners was assessed by calculating Cronbach's alpha and Karl Pearson correlation coefficient and Spearman rank correlation respectively.

***Results:*** The mean time taken for completing the LEFS and LEHRQoL questionnaire was 5 minutes and 2 seconds and 12 minutes and 8 seconds respectively. Internal consistency reliability assessment showed good internal consistency for both the examiners (Cronbach's alpha 0.816 and 0.812). There was a strong positive correlation for the cases (*r* = 0.956, *p* < 0.001; *r* = 0.908, *p* < 0.001) and controls (*r* = 0.992, *p* < 0.001; *r* = 0.985, *p* < 0.001) between the two examiners.

***Conclusions:*** LEFS and LEHRQoL were well accepted among filarial LE patients and the patients with low literacy were able to respond without any difficulty to both assessment tools. LEFS was found suitable for the assessment of lower extremity functions of the LE patients as in other diseases affecting the lower limb and it also indirectly brought out the impact on the QoL.

## Introduction

The most common cause of lymphedema (LE) in developing countries is a preventable parasitic infection caused by *Wuchereria bancrofti*. Lymphatic filariasis (LF) is a chronic parasitic disease of the tropics that leads to significant disability worldwide and it is estimated that 36 million infected people are suffering from chronic manifestations of the disease.^1^ Filarial LE, if left untreated, leads to a chronic stage with intermittent acute episodes of lymphangitis or lymphadenitis affecting patients' capacity to carry out activities of daily life.^2,3^ A majority (95%) of the patients suffer from lower extremity LE and Wijesinghe et al.^4^ has shown that there was a significant association with difficulty in walking.

Global Program for Elimination of Lymphatic Filariasis (GPELF) emphasizes on effective implementation of morbidity management and disability prevention (MMDP) to address the health needs of the filarial LE patients.^5^ Several studies have documented the impact of MMDP on quality of life (QoL) and economic burden resulting from the deformities of the extremities mostly the lower limbs and the recurrent acute dermato lymphangio adenitis episode that the LE patients endure at unpredicted intervals.^6–8^

There are several QoL assessment tools addressing the overall physical, psychological, and social functioning of patients. Though these tools are valuable in assessing the satisfaction of the patients, disease-specific tools are advocated to have a more precious assessment, especially for clinical conditions of arthritis, cardiac disease and posttreatment evaluations. As lower extremities are commonly affected in LF, it is desirable to have a tool that measures the lower extremity functions. The disease-specific functional assessment facilitates the development of appropriate intervention for early recovery. Lower extremity functional scale (LEFS) developed by Binkley^9^ was found to be reliable and easily modifiable in local language without affecting the consistency.^10–13^ This tool was also found to be significantly informative in assessing the lower extremity functions among patients affected by various lower extremities diseases.^14–16^ These studies suggest that LEFS adopted in local language modified to the local situation in patients' vernacular helps to bring out the reliable outcome in lower extremity disorders. The present study was carried out as a part of the LE—Osteoarthritis study to know the feasibility and applicability of the LEFS among filarial LE patients of the lower extremity and to assess its correlation with LE health-related QoL (LEHRQoL) and its Tamil version FLEQoL-T3.

## Materials and Methods

### Study settings

Vector Control Research Centre (VCRC) under Indian Council of Medical Research (ICMR), Pondicherry, India offers MMDP services for filarial LE patients free of cost. LE patients residing in the geographical locations where surveys are carried out for various research projects avail these facilities through monthly follow-up to ensure that LE patients follow home-care procedures. Each patient gets individual attention by the Medical Officer for follow-up advice and those patients with symptoms and signs of acute manifestations will receive conservative treatment until recovery.

### Regulatory approval

The study was approved by the Scientific Advisory Committee (SAC) and the Institutional Human Ethics committee of VCRC, Pondicherry, India (IRB Ref No: IHEC-0118/M).

### Study participants

One of the co-investigators of the project explained the purpose, details of the study, and provided the information sheet in vernacular for better understanding by the study participants. After getting the clarification, if any, the participants provided their written consent to participate. The participants were also aware that they have the liberty to withdraw from the study without assigning any reason without any consequences for their routine MMDP services provided to them. All the participants signed the written informed consent before getting enrolled in the study.

In total, 60 participants, 30 LE patients and 30 persons accompanying the LE patients as controls were recruited in the study following inclusion and exclusion criteria.

### Inclusion criteria

(1) Grade III and IV lower limb filarial LE patients as per WHO grade attending MMDP clinic (2) age between 40 and 65 years (3) both genders (4) LE duration not <5 years (5) body mass index (BMI) up to 40 and (6) individuals willing to give written informed consent for participation were included in the study.

### Exclusion criteria

(1) Known case of rheumatoid arthritis, inflammatory arthritis, systemic lupus erythematosus, polyarthralgia, osteoporotic disease (2) unable to walk without aids, (3) history of fracture of lower limb or spine, any other surgical or medical condition that severely limits the subjects functional (4) blood hemoglobin level <8 gm% were excluded from the study.

### Controls

Individuals accompanying the patients with no history of LE from both genders having BMI up to 40 were included as controls.

### Assessment tools

Two “self-reporting assessment tools” LEFS and LEHRQoL were applied to all participants (patients and controls) after obtaining informed written consent.

### LEFS

Original English version developed by Binkley contains 20 statements to assess the difficulties in carrying out routine activities in, around, and out of the home. As few of these statements do not suit to the local population, first, statements in the English version were modified. Under this exercise, five statements were modified as follows: statement 3, sitting and getting up from bath as sitting and getting up from Indian toilet; statement 5, putting on your shoes and socks as putting on your footwear; statement 10, getting into or out of car as getting into or out of public transport; statement 16, running on even ground as fast walking on even ground; statement 17, running on uneven ground as fast walking on uneven ground.

After modification of the required statements, translation to local vernacular was carried out as follows: The above modified English version of LEFS was translated to Tamil language by one Medical Officer and one Nursing Technical Officer independently. These two independent Tamil versions were back-translated to English by two Technical Officers independently. These two back-translated English versions were checked and corrected independently by one of the investigators for language and alignment of words to retain the meaning of the statements. These two back-translated versions were again translated to Tamil again by the Medical Officer and the most fitting statements from this Tamil version were incorporated in the final Tamil-LEFS. Also, the LEFS was performed independently by two investigators. However, the scoring from the original Binkley version was retained without any modification in Tamil-LEFS version.

### LEHRQoL

The LEHRQoL instrument is a tool to assess QoL of the LE patients and this can be applied as a self-administered tool or through an interviewer. After several modifications and testing the final Tamil version FLEQoL-T3 was applied in the study. This instrument in the form of a questionnaire consisted of four domains, mobility functions, cognitive functions (CogFs), social stigma, and symptom complex altogether with 42 items. These four domains were given 2:2:1:1 weightage to get LE specific QoL score and to compare their correlation with LEFS scores. The mobility and CogFs component contained 14 questions each and stigma and symptoms contained seven questions each. The questionnaire included positive or negative questions for which the respondents choose one appropriate answer from five options and score for each option was precoded. The responses were evaluated on a five-point Likert scale for each response. Response to each item was assigned a score between 0 and 4, with higher scores indicating a best QoL. Each domain score was calculated by adding the individual scores and dividing the total by the number of questions answered. Overall QoL score was calculated by adding the scores of all responses and dividing the total score by the number of responses in all domains. The maximum scores that can be gained were 56, 56, 28, and 28 for mobility, CogFs, stigma, and symptoms respectively and 168 as total LEHRQoL score.

### Data analysis and statistical methods

All data were entered in Microsoft Excel, cross-checked, and transported to SPSS software. Analyses were carried out in SPSS 22.0 version. Mean and standard deviation were computed for age, whereas proportion was calculated for gender, education, income, and BMI Asian category. To assess the feasibility of the LEFS by auxiliary health workers with different educational background and to know the correlation of LEFS with different domains of LEHRQoL, Karl Pearson correlation coefficient and nonparametric Spearman rank correlation was computed. To compare the mean scores of LEFS and LEHRQoL between cases and controls we used student *t*-test. The significant level of 0.05 was considered as acceptable. Internal consistency reliability of LEHRQoL total score and domain-total score measurements were calculated using Cronbach's alpha.

## Results

### Demographics

In total, 30 consecutive eligible LE cases and 30 eligible control participants were included in the study. Characteristics of the participants included in the study are given in Table 1. As seen in the other studies, the proportions of female patients were more, the literacy level was much lower and most of the cases and controls were from the low socioeconomic background. However, it was observed that the proportion of the overweight category was higher among the LE cases and this can be explained by the sedentary lifestyle of the LE patients especially among the female patients in grade III and IV LE.

**Table 1.** Characteristics of the Participants Included in the Study

Variable	LE patients,* n *(%)	Control,* n *(%)
Gender		
Male	7 (23.3)	12 (40.0)
Female	23 (76.7)	18 (60.0)
Education		
Nil	6 (20.0)	8 (26.7)
High school	22 (73.3)	18 (60.0)
Higher secondary +	7 (6.7)	4 (13.3)
Income in Indian Rupees		
≤3000	21 (70.0)	13 (43.3)
3000–6000	5 (16.7)	9 (30.0)
>6000	4 (13.3)	8 (26.7)
Body mass index		
18.50–22.99	1 (3.3)	6 (20.0)
23.00–27.49	15 (50.0)	19 (63.3)
27.50–40.00	14 (46.7)	5 (16.7)
Age (years)		
Mean (SD)	55.6 (10.3)	51.0 (7.2)

LE, lymphedema; SD, standard deviation.

### Schedule feasibility and difficulty level

The study consisted of completion of two assessment tools Tamil LEFS and LEHRQoL—Tamil version FLEQoL-T3 in the form of completing the questionnaire through interview. Analysis of the time taken for completing each of the questionnaires was recorded for both the examiners independently. It was observed that the mean time taken for completing the Tamil-LEFS and FLEQoL-T3 questionnaire was 5 minutes and 2 seconds and 12 minutes and 8 seconds respectively. Patients were able to understand the questionnaire and respond without taking much time. However, two patients were responding slowly and the interviewer had to repeat the questions to complete the questionnaire.

### Lower extremity functions and QoL assessment

Analysis of mean scores for LEFS and four domains of LEHRQoL—FLEQoL-T3 by student *t*-test for equivalence of mean revealed that the total LEFS and total LEHRQoL scores and the total scores of all four domains of QoL were significantly lower than the respective scores of the controls for both the examiners (Table 2). Internal consistency reliability assessment by Cronbach's alpha showed good internal consistency for examiner-1 (Cronbach's alpha 0.816) and for examiner-2 (Cronbach's alpha 0.812), inter-item internal consistency for four domains of LEHRQoL with the total score was found to be at acceptable levels (Table 3).

**Table 2.** Mean LEFS Score and LEHRQoL Score by Different Domains for LE Cases and Controls

Function	Group	*n*	Examiner-1		*p*	Examiner-2		*p*
			Mean	SD		Mean	SD	
LEFS total score	Case	30	56.73	18.06	0.000	58.73	17.51	0.000
	Control	30	72.47	8.58		72.37	8.97	
MoF total score	Case	30	44.23	9.54	0.001	44.57	10.36	0.001
	Control	30	51.53	5.04		51.83	5.11	
CogF total score	Case	30	36.20	15.14	0.000	34.60	17.07	0.000
	Control	30	55.50	1.31		55.13	3.04	
Stigma total score	Case	30	16.07	9.44	0.000	16.33	10.18	0.000
	Control	30	28.00	0.00		28.00	0.000	
Symptoms total score	Case	30	15.47	6.61	0.000	15.40	6.652	0.000
	Control	30	26.73	1.62		26.83	1.76	
QoL total score	Case	30	111.97	34.13	0.000	110.90	36.92	0.000
	Control	30	161.77	6.65		161.80	7.98	

CogF, cognitive function; LEFS, lower extremity functional scale; LEHRQoL, LE health-related quality of life; MoF, mobility function.

**Table 3.** Internal Consistency Reliability of Four Domains of LEHRQoL with Total Score by Examiner for LE Cases (*n* = 30)

Scale/domain	No. of items	Cronbach's alpha	
		Examiner-1	Examiner-2
LEHRQoL total score with all other domains	5	0.816	0.812
LEHRQoL total score with mobility domain	2	0.520	0.501
LEHRQoL total score with cognitive domain	2	0.808	0.839
LEHRQoL total score with stigma domain	2	0.625	0.623
LEHRQoL total score with symptoms domain	2	0.473	0.421

### Assessment of correlations

**FIG. 1.**
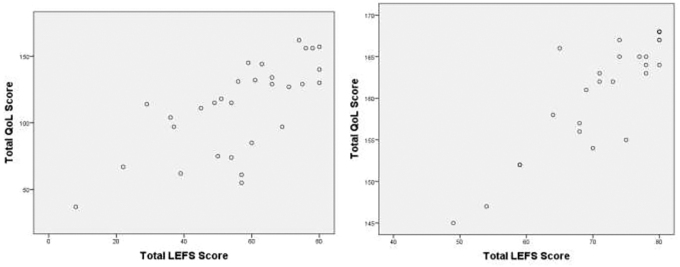
Scatter plot of lower extremity functional scale total score and LE health-related quality of life total score of the LE patients and controls respectively. LE, lymphedema.

Correlation between the two assessment tools LEFS and disease-specific LEHRQoL—FLEQoL-T3 were assessed by scatter plot (Fig. 1). To determine the correlation between the two examiners, Karl Pearson correlation coefficient and Spearman rank correlation were computed for the LEFS score for the cases and controls separately. It was observed that there was a strong positive correlation for the cases (*r* = 0.956, *p* < 0.001; *r* = 0.908, *p* < 0.001) and controls (*r* = 0.992, *p* < 0.001; *r* = 0.985, *p* < 0.001) by the two examiners (Table 4).

**Table 4.** Pearson Correlation and Spearman's Rank Correlation Between Two Examiners for LEFS Total Score

Correlation	Cases (*n* = 30)	Control (*n* = 30)
Pearson Correlation	0.956	0.992
Sig. (two tailed)	0.000	0.000
Spearman's rho	0.908	0.985
Sig. (two tailed)	0.000	0.000

Similarly, based on a series of Karl Pearson correlation coefficient it was observed that there was a good correlation between two examiners for the different components of the LEHRQoL instrument for mobility function (MoF) (*r* = 0.887, *p* = 0.000 and *r* = 0.881, *p* = 0.000); cognitive functions (CogFs) (*r* = 0.949, *p* = 0.000 and *r* = 0.191, *p* = 0.311); social stigma (*r* = 0.845, *p* = 0.000 and *r* = 1.00, *p* = 0.000); and symptoms (*r* = 0.838, *p* = 0.000 and *r* = 0.443, *p* = 0.014) for LE cases and controls respectively. Results of the Spearman rank correlation also indicated that there was a good correlation between the two examiners for MoF (*r* = 0.807, *p* = 0.000 and *r* = 0.970, *p* = 0.000); CogFs (*r* = 0.941, *p* = 0.000 and *r* = 0.596, *p* = 0.001); social stigma (*r* = 0.762, *p* = 0.000 and *r* = 1.00, *p* = 0.000); and symptoms (*r* = 0.834, *p* = 0.000 and (*r* = 0.476, *p* = 0.008) for LE cases and controls respectively (Table 5).

**Table 5.** Pearson Correlation and Spearman's Rank Correlation Between Two Examiners for the Different Domains of Functions and Total LEHRQoL Score

Correlation	Mobility	Cognitive	Stigma	Symptoms	Total LEQoL
LE cases (*n* = 30)					
Pearson correlation	0.887	0.949	0.845	0.838	0.934
Sig. (two tailed)	000	0.000	0.000	0.000	0.000
Spearman's rho	0.807	0.941	0.762	0.834	0.939
Sig. (two tailed)	0.000	0.000	0.000	0.000	0.000
Control (*n* = 30)					
Pearson correlation	0.881	0.191	1.000	0.443	0.797
Sig. (two tailed)	0.000	0.311	0.000	0.014	0.000
Spearman's rho	0.970	0.596	1.000	0.476	0.887
Sig. (two tailed)	0.000	0.001	0.000	0.008	0.000

Karl Pearson correlation coefficient and Spearman rank correlation were computed for cases and control put together and found that there was a good correlation between LEFS total score and the different domains of the LEHRQoL—FLEQoL-T3 scores and total LEHRQoL—FLEQoL-T3 score (Table 6). When similar correlation was computed for LE cases and controls separately it was observed that there was a good correlation between LEFS total score and the different domains of the LEHRQoL—FLEQoL-T3 scores in addition to total LEHRQoL—FLEQoL-T3 score (Table 7).

**Table 6.** Pearson Correlation and Spearman's Rank Correlation Between LEFS Total Score and Different Domains of Functions and Total LEQoL Score (LE Cases and Controls *n* = 60)

Correlation	Mobility	Cognitive	Stigma	Symptoms	Total LEQoL
Pearson correlation	0.923	0.627	0.557	0.687	0.774
Sig. (two tailed)	0.000	0.000	0.000	0.000	0.000
Spearman's rho	0.932	0.631	0.557	0.680	0.795
Sig. (two tailed)	0.000	0.000	0.000	0.000	0.000

**Table 7.** Pearson Correlation and Spearman's Rank Correlation Between LEFS Total Score and Different Domains of Functions and Total LEQoL Score

Correlation	Mobility	Cognitive	Stigma	Symptoms	Total LEQoL score
LE cases (*n* = 30)					
Pearson correlation	0.912	0.488	0.423	0.576	0.700
Sig. (two tailed)	0.000	0.006	0.020	0.001	0.000
Spearman's rho	0.917	0.467	0.420	0.574	0.710
Sig. (two tailed)	0.000	0.009	0.021	0.001	0.000
Control (*n* = 30)					
Pearson correlation	0.874	0.547	1.000	0.481	0.887
Sig. (two tailed)	0.000	0.000	0.000	0.007	0.000
Spearman's rho	0.900	0.339	1.000	0.568	0.863
Sig. (two tailed)	0.000	0.067	0.000	0.001	0.000

## Discussion

**FIG. 2.**
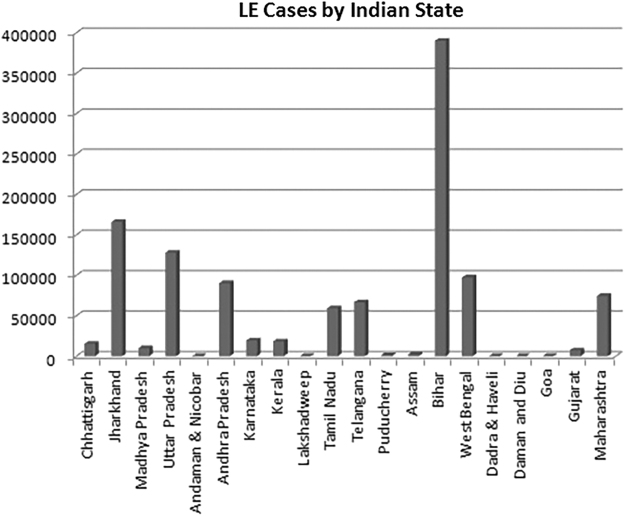
Number of LE cases registered in different states of India during 2017.

Lymphedema affects lower extremity functions to a great extent in both developing and developed countries. In tropical countries, parasitic infections play a major role in the establishment of lower extremities LE. India has the highest number of filariasis cases in the world presenting with an extensive regional difference (Fig. 2). LE, especially in higher grades, has a significant impact on the QoL.^17,18^ However, the impact on lower extremities functions has not been exclusively assessed among filarial LE patients. Though there are several generic questionnaires like Medical Outcomes Study Short Form-36 and HRQoL available, they cannot be considered as the disease-specific measure.^19^ An effective tool is expected to assess the mobility of the lower extremities to assess the real impact on functions. LEFS by Binkley and translated with relevant modifications to local situations has been found to be feasible, cost effective and clinically relevant in the field and acceptable in the communities.^20–22^ LEFS initially applied for outpatients was found to be suitable for the inpatients also.^23^ Several studies have demonstrated the relevance and advantages of applying two assessment tools simultaneously.^24,25^

We included Tamil version LEFS and LEHRQoL disease-specific tool and two examiners applied both these tools among the LE patients and controls. The main focus of this study was to adopt LEFS with modifications required to the local populations and to know the feasibility of its application in the field by the auxiliary health staff with different educational background. In this study, for applying LEFS among the LE patients of Tamil Nadu, South India, very little modifications were required for cross-cultural adaptation before translation. The translated Tamil version LEFS was well received among the LE patients. Auxiliary health staff with different educational background did not find any difficulty in applying the questionnaire among the LE patients of the lower extremities and the inter-observer correlation was very high (*r* = 0.956, *p* < 0.01).

To get a reliable response with cross-cultural adaptation, at least 30 patients needed to be assessed.^26^ We included 30 LE patients and 30 volunteers preferably the persons accompanying the patients for follow-up as controls and found that the response from both groups is reliable. We observed strong correlations for the total LEFS scores between two examiners with different educational background indicating the field applicability of the LEFS tool. The study is also different from other studies in terms of the correlation LEFS score with different domains of the FLEQoL-T3 score developed by the investigators. The responses from the LE patients were found to be reliable as it could clearly distinguish LE patients of the lower extremity from the age-matched controls. We found that the major impacts of lower extremity lymphedema of filarial patients were mobility and stigma domains of the disease-specific LEHRQoL—FLEQoL-T3. Therefore, the-Tamil LEFS is reliable in assessing the functional disability of the lower extremity filarial LE patients and feasible to be applied in the field by the health workers with different educational background.

### Limitations of the study

Major limitation of the study was that we restricted the sample to 30 each in LE cases and controls as the participants have to complete two questionnaires with two examiners leading to the long waiting time for the patients. The other limitation was that the representation of male participants was less compared to female participants affecting the representativeness of all LE patients. In addition, due to the very low literacy level of participants with very few exceptions, these instruments could not be administered as “self-administered tools.”

## Conclusion

In conclusion, the Tamil LEFS required little modification from the original LEFS version and this could bring out the required information from the participants including the filarial LE patients with low literacy level. The study also showed that LEFS and LEHRQoL are well accepted among filarial LE patients and the patients with very low literacy level did not find any difficulty in responding to the statements in both the assessment tools. The 4 domain 42 items LEHRQoL and its Tamil version FLEQoL-T3 instrument had an overall excellent internal consistency.

This is the first study that looked for the correlation between LEFS score and LEHRQoL score and the results showed that there was a good correlation and therefore LEFS is suitable for the assessment of lower extremity functions of the LE patients as in other diseases affecting the lower limb and it also indirectly brings out the impact on the QoL. Tamil LEFS if administered alone, a patient can complete the questionnaire with the interviewer within 15 minutes and therefore LEFS may be utilized as a quick tool to assess the QoL of filarial LE patients of the lower extremity.
